# Evaluation of an International Classification of Functioning, Disability and Health-based rehabilitation for thermal burn injuries: a prospective non-randomized design

**DOI:** 10.1186/s13063-019-3910-6

**Published:** 2019-12-19

**Authors:** Hubert Neubauer, Annette Stolle, Sabine Ripper, Felix Klimitz, Hans Ziegenthaler, Mareike Strupat, Ulrich Kneser, Leila Harhaus

**Affiliations:** 1 Department of Hand, Plastic and Reconstructive Surgery, Burn Center, BG Trauma Center Ludwigshafen, Plastic- and Hand Surgery, University of Heidelberg, Ludwig-Guttmann-Str. 13, 67071 Ludwigshafen, Germany; 2 Moritz Klinik, Hermann-Sachse-Strasse 46, 07639 Bad Klosterlausnitz, Germany

**Keywords:** Rehabilitation, Thermal injuries, Burn injuries, ICF

## Abstract

**Background:**

Severe burn injuries result in relevant restrictions of physical capacity as well as psychological and social integrity and require a specialized rehabilitation. There is a common agreement, among national as well as international burn associations, that burn rehabilitation is a complex, dynamic process which needs an interdisciplinary and specialized treatment team. There is wide agreement that more research is needed in this field.

**Methods/design:**

The aim of the study is to examine the effectiveness and efficiency of our new ICF (International Classification of Functioning, Disability and Health)-based rehabilitation for thermal injuries. Because of ethical reasons, we have chosen a prospective non-randomized design, which takes place at two different rehabilitation centers. At center A, a newly developed ICF-based rehabilitation program was established; at rehabilitation center B, a well-established rehabilitation program has existed for 20 years and is used as reference. The *primary research question* addresses the “Pre-post comparison of the physical and psychological outcome measurements,” *secondary question I* looks at the “Examination of the non-inferiority of the new treatment concept with the established concept,” and *secondary question II* is the “Analysis of the rehabilitation process based on the rehabilitation cycle.”

Only patients of the two burn rehabilitation centers who are insured by workers’ compensation will be asked to participate in this study to avoid outcome bias by insurance status. A physical examination (physical working capacity testing, grip strength, range of motion, and scar evaluation by Cutometer and Vancouver Scar Scale) and a standardized questionnaire battery (Burn Specific Health Scale-Brief , Short Form 36, Impact of Event Scale-Revised, the German version of the Symptom Checklist, the Freiburg Social Support Questionnaire, Patient/Client Satisfaction Questionnaire, Disabilities of the Arm, Shoulder and Hand, and Lower Extremity Functional Scale ) measure physical and psychological conditions. Data will be taken on admission, during stay, and on discharge of the rehabilitation program and at follow-up 3 and 12 months after discharge. A minimum of 162 participants will be enrolled in this clinical longitudinal, prospective, observational study.

**Discussion:**

The proof of the effectiveness of the ICF-based rehabilitation program for thermal injuries will give evidence in a comprehensive way for the first time in this field. As result, a standardized rehabilitation concept will be introduced, which can be provided to other rehabilitation institutions treating thermal injuries.

**Trial registration:**

German Clinical Trials Register, DRKS00017702. Registered on 2 September 2019.

## Background

Progress in the medical and surgical management of major burn injuries has significantly increased the likelihood of survival, even in those seriously injured. Since survival from large burns has become more common, mortality is no longer the only relevant outcome measure [1]—functional outcome and quality of life have become more important [2, 3]. Burn injuries result in impaired physical capacity and physical and social integrity and may have a life-long impact on both psychological and physical functioning [4]. The aim of a burn rehabilitation program is to resolve physical and psychological recovery and return into social life and the workplace. During the rehabilitation process, the injured persons and those in their social environment should accept and learn an unbiased handling of the consequences of the injury. These goals can be achieved by helping the person to regain activity and providing behavioral therapy, compensatory strategies (functional adaptation), and assistance in coping with the trauma [5, 6].

There is strong agreement that rehabilitation of patients after burn injuries requires a multidisciplinary team. An increasing number of publications are addressing the relevance of rehabilitation after burn injuries, but structured clinical studies are still rare. Existing studies mostly assess only one special aspect of the treatment program [7–10] or one affected domain such as scarring, quality of life, or mental health [11, 12]. References to the International Classification of Functioning, Disability and Health (ICF) system, a framework which incorporates physical, emotional, environmental, and social aspects of functioning, are rare. In addition to the small overall number of studies, comparison of results and populations is difficult since there are no clear standards for documentation [5, 11].

National and international burn associations provide health care professionals with guidelines for burn rehabilitation [13]. Besides differences in elaborateness, they all confirm the importance of this phase in the treatment of burn injuries. The most clearly defined indications for burn rehabilitation are described in the guidelines of the German Society for Burn Medicine, as listed in Table 1 [6, 14].

**Table 1:** Indications for burn rehabilitation

Main indications	Burns degree II ≥ 20% TBSA
	Burns degree III ≥ 10% TBSA
	Burns of face, hands, feet, or genital area
	Scarring with significant limitations of the large joints
Further indications	Remaining functional neurological deficit
	Functional deficits after high-voltage accident
	Limb loss
	Lasting loss of condition and strength
	Problematic psychosocial situation
	Psychiatric disorders after trauma (adjustment disorders, post-traumatic stress reactions, phobic reactions)
	Age more than 50 years
	Thermal accidents in childhood

*TBSA* Total body surface area

Rehabilitation is a comprehensive process involving a multidisciplinary team working in collaboration to optimize patients’ recovery. Main elements of the treatment after burn injuries are rehabilitative nursing for skin and wound care, physical, occupational, and sports therapy, and psychotherapy [15]. Additional support by social workers is needed to advise the patient on the benefits of social security funds and to facilitate the return to work by making contact with the employer and human resources. During the rehabilitation process, consultations involving multiple other medical disciplines such as pain medicine are often needed. Regular meetings of a multidisciplinary team focusing on the quality of patients’ lives are recommended [6].

In rehabilitation center A (BG Trauma Center Ludwigshafen) a new ICF-based specialized burn rehabilitation program was elaborated in 2012 and established in 2014. As a control, the elements of the program and the patients’ outcomes will be compared to those of the well-established burn rehabilitation program of rehabilitation center B. Center B’s program concept was established in 1999 and also includes ICF-based elements. The purpose of the study is to establish standards and evaluate the effectiveness of the programs to meet the requirements of evidence, quality assurance, and cost-effectiveness in the treatment of such complex and costly injury patterns.

### International Classification of Functioning, Disability and Health

**Fig. 1:**
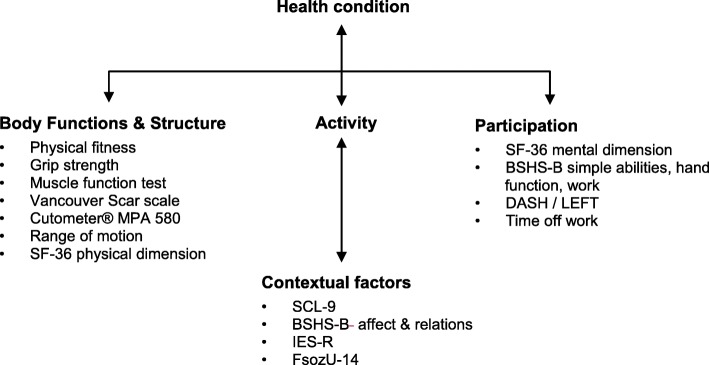
Interactions between ICF components (according to WHO [16]) and associated outcome measures used in the present study

We have chosen the outcome measures along the ICF concept, since it is a highly useful framework describing and organizing health conditions in a comprehensive way involving a biopsychological model. Furthermore, it provides a standard language and a conceptual basis for the definition and measurement of health and disability. Since the ICF was introduced by the World Health Organization (WHO) in 2001 [16], it has become the most used instrument to describe deficits of functional health conditions, disability, social impairment, and relevant environmental factors. In order to provide a global picture of the patients’ status as accurately as possible, we have chosen outcome measures from the main domains of the ICF concept (see Fig. 1):

- Body function and structure
- Activity and participation
- Contextual factors (composed of environmental and personal factors).

## Aim of study

The aim of the present study is to evaluate the effectiveness of the rehabilitation programs for thermal injuries at two centers in Germany. Changes in health status are measured along the ICF constructs of body function and structure, activities, participation, and contextual factors (environmental and personal factors) in order to address health issues as completely as possible. Patients’ conditions in the different domains will be measured before and after the rehabilitation and at two follow-up points, 3 and 12 months after rehabilitation, to quantify long-term changes and treatment effects. Further, the process of rehabilitation will be looked at very closely regarding goal setting and attainment. Patients‘ and therapists‘ satisfaction with the rehabilitation progress will be requested on a regular weekly basis. From the close observation of the rehabilitation process we expect to get a deeper understanding of the needs of our patients and a basis to improve the treatment concept if necessary.

## Methods

### Ethics approval and consent to participate

The study is approved by the ethics committee of Rheinland-Pfalz, Mainz, Germany. We ask potential participants for written consent prior to their inclusion in the study.

### Participants

The participants are admitted to the two burn rehabilitation centers in Germany dependent on proximity to their place of residence and availability of a contemporary treatment capacity. Preliminary analyses revealed an overall similarity between the both programs except for small differences: The rehabilitation program in center A contains more occupational therapy; at center B the program contains additional balneotherapy, which involves treatment of the skin by bathing in natural mineral water. Center A is located at the campus of a level 1 trauma center, whereas center B is located in a health resort. The duration of the rehabilitation is planned for 3 weeks in both facilities and can be extended individually.

Only patients of the two burn rehabilitation centers who are insured by workers’ compensation (*Deutsche Gesetzliche Unfallversicherung*) are asked to participate in this study to avoid outcome bias by insurance status. This approach is described in other studies for work-related burn injuries [11] or other orthopedic injuries [17]. This effect is described in terms of satisfaction, pain, functional measures, return to work rate, and adverse events [18].

To start a rehabilitation program, the acute treatment must be completed, and no surgical interventions should be planned during the rehabilitation period. Mostly stable skin and scar conditions are essential to initiate a multimodal treatment to support scar maturing. In case of residual defects, healing under conservative treatment should be expected within a short time. An additional requirement is a sufficient cardio-pulmonary exercise capacity. Table 2 presents the inclusion and exclusion criteria for the study.

**Table 2:** Inclusion and exclusion criteria for the present study

Inclusion criteria	Written consent
	Sufficient knowledge of German in speech and writing
	Age ≥ 18 years Work-related accident
Exclusion criteria	Serious cognitive impairment (e.g., advanced dementia, acute psychosis, traumatic brain injury)
	Declining of the study

Data will be collected on prepared case report forms (CRFs) and by questionnaires and will be entered continuously into an Access database.

### Interventions

No interventions besides the standard rehabilitation program in both centers are planned.

### Objective

The aim of the study is to evaluate the newly established rehabilitation concept in terms of pre-post comparison and through analyzing the rehabilitation process in matters of patients’ satisfaction with goal attainment.

#### Primary research question

Do the patients benefit from the rehabilitation program regarding their physical and psychological health conditions? Furthermore, are the changes steady after rehabilitation?

##### Hypotheses

The alternative hypothesis (H1) is as follows:

- Physical and psychological outcome measures are enhanced after burn rehabilitation compared to measures before rehabilitation.
- Physical and psychological outcome measures are enhanced 3 and 12 months after rehabilitation compared to measures before rehabilitation.

The null hypothesis (H0) is as follows:

- Physical and psychological measurements are not improved after the burn rehabilitation compared to measures before rehabilitation.
- Physical and psychological outcome measures are not enhanced 3 and 12 months after rehabilitation compared to measures before rehabilitation.

#### Secondary research questions I and II

##### Secondary research question I

Are there any differences regarding the effect of the rehabilitation treatment on the outcome measures between the two centers (non-inferiority of center A to center B)?

##### Secondary research question II

What are the most common rehabilitation goals of the patients, and to which ICF categories are they primarily linked? How do goals change during the course of rehabilitation? And how satisfied are the patients’ with their goal attainment?

### Design

This study uses a quasi-experimental prospective longitudinal design, including treating patients at two burn rehabilitation centers in Germany. For ethical reasons, randomization of patients or treatments is not possible; therefore, we added the non-inferiority question (comparison of the two centers). No intervention besides the standard rehabilitation program in both centers is planned. Participants will be screened at admission, at discharge, and at follow-up 3 and 12 months after discharge.

## Outcomes

### Initial patient characteristics

The patient demographics measured include age, gender, race/ethnicity, family status, and employment status. Medical characteristics include diagnosis and therapies, total body surface area (TBSA), partial thickness and full thickness burns, Abbreviated Burn Severity Index (ABSI), inhalation trauma, pneumonia or sepsis, other diagnoses, and therapies.

### Outcome measures

The types of outcome measures used to collect participant data are listed in Table 3.

**Table 3:** Data collected at baseline (T1) and follow-up

	T1 Admission	Weekly	T2 Discharge	T3 3 months after discharge	T4 12 months after discharge
Physical outcome measures					
PWC 150	X		X	X	X
VSS	X		X	X	X
ROM of the large joints	X		X	X	X
Cutometer	X		X	X	X
Grip strength	X		X	X	X
DASH	X		X	X	X
Muscle function	X		X	X	X
LEFS	X		X	X	X
Psychological outcome measures					
BSHS-B	X		X	X	X
SF-36	X		X	X	X
IES-R	X		X	X	X
SCL-K-9	X		X	X	X
F-SozU	X		X	X	X
CSQ-8			X	X	X
ICF-based rehabilitation goals	X	X			
Semi-structured interview			X		

*PWC* physical working capacity test, *VSS* Vancouver Scar Scale, *ROM* range of motion, *DASH* Disabilities of the Arm, Shoulder and Hand, *LEFS* Lower Extremity Functional Scale, *BSHS-B* Burn Specific Health Scale-Brief, *SF-36* Short Form Health Survey, *IES-R* Impact of Event Scale-Revised, *SCL-K-9* Symptom Checklist 9, *F-SozU* Freiburg Social Support Questionnaire, *CSQ-8* Patient/Client Satisfaction Questionnaire, *ICF* International Classification of Functioning, Disability and Health

#### Physical outcome measurements

##### Physical fitness

General physical capacity is measured by the physical working capacity test 150 (PWC 150). The participant sits on a bicycle ergometer (Ergo-Fit Cardio Line 3000; Sport-Tec Physio & Fitness, Pirmasens, Germany). Heart rate is measured with a heart rate monitor with chest strap (Sigma Model Classic; Sigma-Elektro, Neustadt/Weinstraße, Germany) and will be recorded before, during, and after the test. The participant should reach a pedal-revolution rate of 70 rounds per minute (rpm). The exercise test begins with a workload of 25 watts (W), and the software automatically increases the workload by 25 W every 2 min. The target heart rate (beats/min) is calculated as 150 minus the age of the participant. When the individual target heart rate has been exceeded for more than 30 s, the final workload stage is reached and will be recorded. After this stage, a 5-min recovery period at 25 W will follow in which the heart rate recovery is recorded. The applied termination criteria are decreasing heart rate despite increasing exertion (for more than 30 s), chest pain or a feeling of constriction in the chest, shortness of breath, headache, dizziness, subjective exhaustion, leg fatigue, leg cramps, and a pedal-revolution rate below 60 rpm.

##### Grip strength

Grip strength will be measured with a Jamar dynamometer (Sammons Preston Inc., Bolinbrook, IL, USA) at level 2. The participant will sit in front of the rater, holding the elbows close to the body and flexed at 90 degrees with the wrists in a neutral position. The participant will be requested to press the dynamometer with maximum force. Three trials with each hand will be carried out [19].

##### Muscle function test

Major muscle function is tested according to Janda [20]. Muscles of the upper and lower extremities are tested by a skilled physiotherapist, rating the muscle functioning from 0 = “no contraction” to 5 = “normal functioning.”

##### Burn scar assessment

Burn scars will be assessed at two representative sites using the Vancouver Scar Scale (VSS) and cutometry. Both sites are selected by the examiner as representative and are documented by an anatomical description of the location and by photo documentation to ensure retrieval of exactly the same spot in the follow-up measures.

The VSS will be used to clinically evaluate the burn scar formation and characteristics. The VSS assesses four parameters of the scar, including vascularization, pigmentation, pliability, and height/thickness, giving a range of 0–14 in the total score. The appearance of the burn scar will be evaluated by a physician [21].

Properties of the scar will be measured objectively with the Cutometer® Multi Probe Adapter (MPA) 580 (Courage + Khazaka electronic GmbH, Cologne, Germany). The Cutometer® MPA 580 is a device that measures the firmness and elasticity of skin. A negative pressure is created with a vacuum, the skin is drawn into the aperture of the probe, and after a defined time it is released. Inside the probe, the height of skin that is drawn up is determined by a non-contact optical measuring system. The resistance of the skin to the negative pressure (firmness) and its ability to return to its original position (elasticity) are displayed as curves (penetration depth in millimeters/time), and parameters (R0 and R2) are calculated [22].

##### Burn contracture

The extent of burn contracture of the large joints will be measured by range of motion (ROM) using a standard goniometer.

##### Self-reporting questionnaires

Two questionnaires are used. The Disabilities of the Arm, Shoulder and Hand (DASH, German version [23]) questionnaire is a 30-item self-report outcome measurement for physical function and symptoms in patients with one or multiple musculoskeletal disorders of the upper limb. In addition to the Disability/Symptom module (30 items, scored 1–5), the DASH contains two optional modules: Sports/Performing Arts and Work (each scale consists of four items, scored 1–5). Each score is converted to a 0–100 scale. Higher scores indicate greater disability. The DASH is widely used and has demonstrated its clinical value for several different medical conditions of the upper limb.

The Lower Extremity Functional Scale questionnaire for patients with foot and/or leg injuries (LEFS [24]) is a 20-item condition-specific functional status measure applicable to a wide spectrum of patients with lower extremity conditions of musculoskeletal origin. Each LEFS item is scored on a Likert scale from 0 to 4, with higher scores representing higher functional levels; the maximum score is 80. Normative data are available for comparison.

#### Psychological outcome measurements

##### Burn Specific Health Scale-Brief questionnaire (BSHS-B, German version [25])

The BSHS-B measures the most important domains of a patient’s quality of life that are affected after burn injury. The BSHS-B consists of 40 items divided into 9 different subscales: affect, interpersonal relationships, sexuality, simple abilities, hand function, work, heat sensitivity, treatment regimens, and body image. Each item asks about the extent of a specific limitation and can be answered on a 5-point Likert scale that ranges from 0 = “extremely limited” to 5 = “not limited at all.” The higher the score, the lower the perceived impairment.

##### Short Form Health Survey (SF-36, German version [26])

The SF-36 questionnaire is one of the most commonly used assessments to measure an adult person’s perceived physical and mental health-related general quality of life. The questionnaire includes eight scales; four can be summarized into the General Mental Health scale, while the remaining four domains form the General Physical Health scale. All subdomains are assessed using a 5-point Likert scale (except Physical Functioning, which is evaluated using only a 3-point scale). The scores are converted to a 0–100 scale. The higher the score, the higher the perceived quality of life.

##### Symptom Checklist-9 (SCL-K-9 [27])

The original Symptom Checklist-90-Revised (SCL-90-R) is a widely used questionnaire developed to determine a number of psychological symptoms. Its shortened version has shown equally high internal consistencies as a suitable screening instrument to assess a wide range of psychopathologic symptoms. The questionnaire contains one-dimensional items which are scored on a 4-point Likert scale ranging from 1 (“not at all”) to 4 (“extremely”).

##### Impact of Event Scale-Revised (IES-R [28])

The IES-R is a 22-item, self-report questionnaire that assesses post-traumatic stress symptoms in relation to a specific event. It is one of the most commonly used metrics of post-traumatic stress disorder (PTSD) symptomatology. Items are rated on a 5-point scale ranging from 0 (“not at all”) to 4 („extremely”).

##### Recording perceived social support (F-SozU-14 [29])

A person’s perceived social support will be measured with the short version of the Freiburg Social Support Questionnaire (F-SozU-14), a 14-item questionnaire comprising emotional and practical support as well as social integration and social burdens. The items are rated on a 5-point Likert scale, ranging from 1 (“does not apply”) to 5 (“exactly applicable”).

##### Patient/Client Satisfaction Questionnaire (CSQ-8 [30])

At discharge and after 3 and 12 months, patients are asked to complete the German version of the CSQ-8. The CSQ-8 is a unidimensional, 8-item questionnaire assessing patient satisfaction with the rehabilitation. The items are rated on a 4-point Likert scale, ranging from 1 to 4. A higher score indicates higher satisfaction with the rehabilitation program.

##### Rehabilitation goals

Participants are questioned at the beginning of the rehabilitation about the primary goals they want to reach during the course of rehabilitation. Once a week they as well as the therapists are asked to fill out a questionnaire about their satisfaction with goal achievement. Further, they can express if their goals for the following week have changed. These formulated goals will be linked to the ICF categories.

### Semi-structured feedback interview

At the end of the rehabilitation program, a semi-structured feedback interview will be held in which the patients will be asked to describe their experiences with the rehabilitation program by a psychologist who was not involved in the rehabilitation process.

#### Statistical considerations

##### Sample size

We calculated the required case number for paired unilateral *t* tests for pre-post comparisons. Due to the small time intervals between the measurements, a small-to-medium effect size of 0.25 with an α-error probability of 0.05 is expected. An expected correlation of the measured values of at least 0.60 requires a power of 0.80. The power analysis revealed a sample size of 162 patients, *N* = 81 per center. Assuming a drop-out rate of 20%, as usually expected in a clinical trial, 92 patients per center would need to be enrolled.

##### Interim analysis and stopping rules

After 2 years as a first milestone, a preliminary evaluation is planned. Termination of the study is considered in the event that severe side effects of the rehabilitation are shown. This includes both physical and psychological aspects. A significant failure in reaching the planned sample size is also defined as a termination criterion.

##### Randomization

No randomization is planned because of ethical reasons. The assignment of a patient to center A or B depends solely on the proximity of the patient’s hometown and the capacity of the center.

##### Statistical methods

Data will be collected continuously. For the statistical analysis, data will be checked for a normal distribution. Data are expressed by means with standard deviations and medians with interquartile range as appropriate. Categorical variables will be expressed as percentages.

Pre-post comparison (T1–T2) of the outcome measures will be tested by paired one-sided *t* tests. Longitudinal treatment effects will be tested by paired one-sided *t* tests between T1 and T3 and between T1 and T4.

The non-inferiority between the two centers will be proved with a power of 0.80 if differences are smaller than 0.4 standard deviation. Since randomized assignment to the centers is not possible, a potential selection bias between the samples will be matched by age, gender, TBSA, ABSI, and inhalation trauma. In case of an imbalance of the two samples, the analysis will be made along the procedure of propensity score matching.

A descriptive analysis of the patients’ rehabilitation goals and their linkage to the ICF categories will be performed. Patients’ and therapists’ assessments of goal achievement will be compared. The mean frequency of changing goals during the course of rehabilitation will be obtained. Furthermore, patients’ satisfaction with rehabilitation will be assessed with a qualitative interview, which will be evaluated by content, and the results of the CSQ-8 will be expressed by means and standard deviations.

The statistical evaluation will be carried out in cooperation with the statistician who participated in the design of the study (see Acknowledgements).

#### Timeframe of the study

A total of 4 years is planned for the realization of this study. Measurements are taken on admission, at the end of the rehabilitation program, and on follow-up 3 and 12 months after discharge. Enrollment in the study ends after 3 years.

## Discussion

The primary focus of this study is to evaluate the rehabilitation program of two different burn rehabilitation centers. Since there is consensus in the burn literature that outcome is multidimensional for burn survivors, we included a broad spectrum of outcome parameters for the pre-post comparison. We chose the outcome measures along the ICF concepts of body function and structure (exercise capacity, muscle function, scar assessment), personal and environmental factors (mental health, social support), and activities and participation (simple activities, activities of daily living, return to work) in order to get a holistic view about the impact of burn injuries and the treatment necessities.

The instruments we chose are predominantly burn-specific (e.g., the BSHS-B) or frequently used in other burn injury studies (e.g., the SF-36 and IES-R) to enable better comparison with other studies. Instruments used for the evaluation of body function and structure are standard assessments such as the PWC, muscle function, and grip strength or commonly used instruments for scar assessment like the VSS und the Cutometer® MPA 580. The assessment will be conducted before and after the rehabilitation program and at two follow-up points (3 and 12 months after rehabilitation). The long follow-up period was chosen to prove the long-term effect of the rehabilitation program on the wellbeing of the patients. In the best case, patients will be enabled to fully participate again in their normal life in all aspects through the rehabilitation treatment. Since the complex multidisciplinary rehabilitation treatment is very strenuous for the patients as well as very cost intensive for the insurance, it is also essential to prove its effectiveness and quality to avoid unnecessary strain on the patients as well as unnecessary costs. After completion of the study, it is further planned to integrate the assessment instruments into clinical routine, if they prove to be relevant, and to provide these standardized documents to other interested rehabilitation facilities for burn injuries in Germany.

The quasi-experimental prospective pre-post design was chosen due to ethical reasons, which do not allow a classical randomization. To balance this methodological limitation, we implemented the non-inferiority design between the two centers. The rehabilitation programs of the two centers were established independently from each other, at different points of time, but both are multidisciplinary ICF related and include the treatments recommended by national and international guidelines. Therefore, we do not hypothesize any difference in treatment effectiveness between the two centers.

A core element of our study is the assessment of patients’ rehabilitation goals, their possible change over the course of the rehabilitation treatment, and the patients’ satisfaction with goal achievement. The detailed observation of the rehabilitation process is one of the strengths of our study. We expect the ICF goals to change during the rehabilitation process from activities of daily living to more complex activities as well as work-related and social items. Assessment of patients’ rehabilitation goals and their satisfaction with goal achievement will also be an important factor for evaluation of our rehabilitation program. Rehabilitation studies in other domains show that a patient-centered, ICF-related program will have the most positive effect on the patients [31]. Furthermore, the rehabilitation goals of the patients will select the most relevant ICF categories and allow an adaption of the rehabilitation program. To our knowledge, no similar studies with an adult population exist until now in the literature.

We only include participants insured by worker’s compensation in order to avoid an outcome bias due to insurance status. This limits the possibility to generalize the results to patients of other rehabilitation centers or those with other types of insurance. However, we do not expect differences between our sample and patients of other burn rehabilitation centers in Germany.

The effect of rehabilitation is analyzed by a multidisciplinary team consisting of psychologists, physiotherapists and occupational therapists, burn specialists, and rehabilitation physicians. Altogether the findings will provide a better understanding of the rehabilitation process and its effects after burn injury. The detailed view on the impairments and rehabilitation goals on the basis of the ICF concept will enable us to determine if all affected dimensions are addressed by the treatment and therefore if the rehabilitation program supports patients’ recovery at its best.

### Consolidated Standards of Reporting Trials (CONSORT) and Standard Protocol Items: Recommendations for Interventional Trials (SPIRIT) Statements

The authors hereby declare that the final report will follow the CONSORT guideline. The SPRIT checklist is provided as Additional file 1.

### Trial status

The trial protocol is version 7.0, dated 08/18/2017.

Recruitment started in November 2018 and is expected to be completed in November 2021.

## Supplementary information

**Additional file 1.** SPIRIT 2013 checklist: recommended items to address in a clinical trial protocol and related documents.

## Data Availability

Not applicable.

## Abbreviations

ABSI: Abbreviated Burn Severity Index
ADL: Activity of daily living
ASD: Acute stress disorder
BSHS-B: Burn Specific Health Scale-Brief
CRF: Case report form
CSQ-8: Patient/Client Satisfaction Questionnaire
DASH: Disabilities of the Arm, Shoulder and Hand
F-SozU: Freiburg Social Support Questionnaire (recording perceived social support)
ICF: International Classification of Functioning, Disability and Health
IES-R: Impact of Event Scale-Revised
LEFS: Lower Extremity Functional Scale
LOS: Length of stay
PTSD: Post-traumatic stress disorder
ROM: Range of motion
SF-36: Short Form Health Survey 36
TBSA: Total body surface area
VSS: Vancouver Scar Scale
