# Supplementary material for: Evaluation of an International Classification of Functioning, Disability and Health-based rehabilitation for thermal burn injuries: a prospective non-randomized design
Source: Trials. 2019 Dec 19;20:752. doi: 10.1186/s13063-019-3910-6 (PMC6923835; doi:10.1186/s13063-019-3910-6)
Supplement: Supplementary file 1 — Additional file 1. SPIRIT 2013 checklist: recommended items to address in a clinical trial protocol and related documents. [file 13063_2019_3910_MOESM1_ESM.docx]

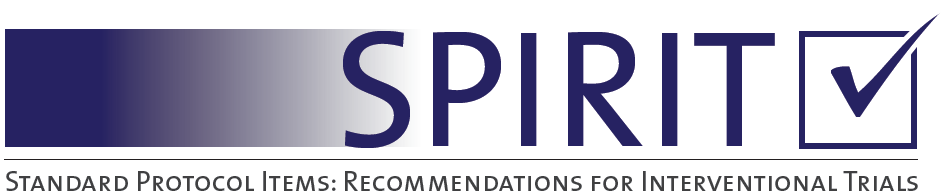


SPIRIT 2013 Checklist: Recommended items to address in a clinical trial protocol and related documents*

| Section/item | ItemNo | Description |
| --- | --- | --- |
| **Administrative information** | | |
| Title | 1 | Evaluation of an ICF-based Rehabilitation Concept for Thermal Injuries |
| Trial registration  (page 11 of the manuscript) | 2a | Deutsches Register Klinischer Studien (DRKS)  German Clinical Trials Register  <https://www.drks.de/drks_web/navigate.do?navigationId=search&reset=true> |
|  | 2b | DRKS00017702 |
| Protocol version  (page 11 oft the manuscript) | 3 | 18.08.2017 Version 7.0 |
| Funding  (page 11 oft the manuscript) | 4 | Deutsche Gesetzliche Unfallversicherung e. V.,  Glinkastraße 40,  10117 Berlin  Germany |
| Roles and responsibilities  (page 11 of the manuscript) | 5a | Prof. Dr. med. Leila Harhaus  Senior Consultant and senior trial leader  Department of Hand, Plastic and Reconstructive Surgery  - Burn Center -  BG Trauma Center Ludwigshafen  Plastic and Hand Surgery  University of Heidelberg  BG - Unfallklinik Ludwigshafen  Ludwig-Guttmann-Str. 13  67071 Ludwigshafen  Germany  Dr. Annette Stolle  Department of Hand, Plastic and Reconstructive Surgery  - Burn Center -  BG Trauma Center Ludwigshafen  Plastic and Hand Surgery  University of Heidelberg  BG - Unfallklinik Ludwigshafen  Ludwig-Guttmann-Str. 13  67071 Ludwigshafen  Germany  Dr. Hubert Neubauer  Department of Hand, Plastic and Reconstructive Surgery  - Burn Center -  BG Trauma Center Ludwigshafen  Plastic and Hand Surgery  University of Heidelberg  BG - Unfallklinik Ludwigshafen  Ludwig-Guttmann-Str. 13  67071 Ludwigshafen  Germany  Dr. Sabine Ripper  Department of Hand, Plastic and Reconstructive Surgery  - Burn Center -  BG Trauma Center Ludwigshafen  Plastic and Hand Surgery  University of Heidelberg  BG - Unfallklinik Ludwigshafen  Ludwig-Guttmann-Str. 13  67071 Ludwigshafen  Germany  Felix Klimitz  Department of Hand, Plastic and Reconstructive Surgery  - Burn Center -  BG Trauma Center Ludwigshafen  Plastic and Hand Surgery  University of Heidelberg  BG - Unfallklinik Ludwigshafen  Ludwig-Guttmann-Str. 13  67071 Ludwigshafen  Germany  Mareike Strupat  Department of physical and occupational therapie  BG Trauma Center Ludwigshafen  BG - Unfallklinik Ludwigshafen  Ludwig-Guttmann-Str. 13  67071 Ludwigshafen  Germany  Dr. Hans Ziegenthaler  Burn Rehabilitation Department  Moritz-Klinik Bad Klosterlausnitz  Department of Rehabilitation and Regenerative Medicine  Hermann-Sachse-Strasse 46,  07639 Bad Klosterlausnitz  Germany  LH, SR and AS conceived of the study. LH, SR, AS, HN and HZ initiated the study design and FK and MS helped with implementation. UK supervised the design of the study and the study protocol. LH and SR are grant holders. LH is the senior leader of the trial and have main responsibility at the BG Trauma Center in Ludwigshafen, HN is the junior trial leader. HZ is the senior trial leader at the Moritz-Klinik. AS is conducting the primary statistical analysis. All authors contributed to refinement of the study protocol. |
|  | 5b | Deutsche Gesetzliche Unfallversicherung e. V.,  Glinkastraße 40,  10117 Berlin  Germany  Projectnumber: FF-FR 0268 |
|  | 5c | The development-process of the study design was supervised by a committee including experts, contributors of the sponsor and representative of burn patients.  Study sponsor is not involved in collection, management, analysis, and interpretation of data; nor of writing of the report or in the decision to submit the report for publication. They will be annually informed about the progress of the study. |
|  | 5d | The coordinating center organizes and supervises the process of data collection and data evaluation. Compliance with the schedule and patient enrollment is monitored biannually and reported to the study sponsor. Annually the participating institutions and a committee of experts (including extern experts, contributors of the sponsor and representative of burn patients) meet to report and accompany the study. |
| Introduction |  |  |
| Background and rationale  (page 3 of the manuscript:  Background) | 6a | Aim of the study is to examine effectiveness and efficiency of a new ICF (International Classification of Functioning, Disability and Health)-based rehabilitation concept.  Burn injuries may have a life-long impact on psychological and physical functioning (Spronk 2018). High quality rehabilitation should minimize the negative burden of impairment and ensure the best possible outcome for patients in terms of participation and reintegration. Although there is a strong agreement in healthcare professionals about the necessity for a multidisciplinary rehabilitation team and about the treatment contents (Paprottka et al. 2016), there is a lack of studies evaluating these programs. However, evaluating the effectiveness of such programs is essential to meet the requirements of evidence, quality assurance and cost-effectiveness in the treatment of complex injury patterns.  The applied treatment elements are rehabilitative nursing for skin and wound care, physical therapy, occupational therapy, sports therapy and psychotherapy (Richard 2009). These elements are strongly recommended in the national guidelines for burn rehabilitation in Germany (Ziegenthaler 2005). No new or untested interventions are applied.  Since rehabilitation after burn injury is essential for prevention of hypertrophic scarring and contractures, a randomized control study would do harm to the control subjects. Therefore, we chose a weaker prospective pre-post and follow-up design and the non-inferiority test between two independent rehabilitation centers. |
| (page 4 of the manuscript:  Aim of the Study) | 6b | In rehabilitation center A a new ICF-based specialized burn rehabilitation program was established. The patients’ outcome will be compared to the well-established burn rehabilitation program of rehabilitation center B. Its concept was established in 1999 and includes also ICF-based elements. |
| Objectives  (page 4 of the manuscript:  Aim of the Study) | 7 | Physical and psychological outcome measures are enhanced after burn rehabilitation and 3 and 12 months after rehabilitation to measures before rehabilitation.  We expect no significant differences regarding the effect of the rehabilitation treatment on the outcome measures between the two centers (non-inferiority).  What are the most common rehabilitation goals of the patients, and to what ICF categories they are mainly belonging? How are goals changing during rehabilitation? And how is patients’ satisfaction with goal attainment? |
| Trial design  (page 6 of the manuscript:  Design) | 8 | The trial is designed as a prospective clinical non-inferiority trial. Due to ethical reasons it is not possible to refuse rehabilitation after severe trauma. That’s why we compare the new rehabilitation program of center A with the well-established program of center B. Allocation to the study sites depends on proximity of residence and availability of treatment capacity. |
| Methods: Participants, interventions, and outcomes | | |
| Study setting  (page 5 of the manuscript:  Participants) | 9 | Patients at two burn rehabilitation centers in Germany are included. Center A is located at the campus of a level 1 trauma center, whereas center B is located at a health resort. (List of study sites can be requested from the investigators.) |
| Eligibility criteria  (page 3 of the manuscript:  Background and  Page 5:  Participants) | 10 | The participants are admitted to the two burn rehabilitation centers in Germany dependent on proximity of residence and availability of a contemporary treatment capacity.  Indications for burn rehabilitation:  Main indications   - Burns degree II° ≥ 20% TBSA - Burns degree III° ≥ 10% TBSA - Burns of face, hands, feet or genital area - Scarring with significant limitations of the large joints   Further indications   - remaining functional neurological deficit - functional deficits after high-voltage accident - Limb loss - lasting loss of condition and strength - problematic psychosocial situation - psychiatric disorders after trauma (adjustment disorders, post-traumatic stress reactions, phobic reactions)   Inclusion criteria:   - written consent - sufficient skills in German language - age ≥18 years - Work-related accident   Exclusion criteria:   - Serious cognitive impairment (eg. advanced dementia, acute psychosis, traumatic brain injury) - Declining of the study   At both study sites treatment of patients after burn injuries has a long tradition. Leader of the departments are medical specialists for rehabilitative medicine and members of the German society of burn medicine. In both rehabilitation teams physiotherapists, occupational therapists, nurses, psychologists, social workers and a health professionals are working together. They all have long experience (several years) in burn specific treatment. |
| Interventions  (page 5 of the manuscript:  Interventions)  (page 9 of the manuscript:  Sample size)  (page 5 of the manuscript:  Participants)  (page 5 of the manuscript:  Interventions) | 11a | No intervention besides the standard rehabilitation program in both centers is planned.  Both rehabilitation programs have a multidisciplinary team approach. Main elements are physical therapy, occupational therapy, sports therapy and psychotherapy. Rehabilitative nursing for skin and wound care is carried out if necessary. Additional support by social workers and consultations of multiple other medical disciplines such as pain medicine are offered if needed. Meetings of a multidisciplinary team focusing on the quality of patients’ lives are held weekly. The rehabilitation program in center A contains more occupational therapy; at center B the program contains additional balneotherapy. Center A is located at the campus of a level 1 trauma center, whereas center B is located at a health resort. The duration of the rehabilitation is planned in both facilities with 3 weeks and can be extended individually. |
|  | 11b | Criteria for discontinuing is defined if serious side effects of the treatment are found such as severe somatic problems, a worsening of the wound and scarring situation or the extent of movement due to the treatment as well as psychological side effects such as psychological destabilization. A significant failure of the planned sample size would also be a termination criterion. |
|  | 11c | Both rehabilitation programs are carried out as inpatient measures, so that participation is continuously documented and monitored. |
|  | 11d | Participation in other studies is not allowed. No restriction of relevant concomitant care and interventions are made due to the study. |
| Outcomes  (page 6 – 8 of the manuscript:  Outcome measures) | 12 | Primary outcome of the study is the pre-post and follow-up comparison for variables representing parts of the three main domains of the ICF concept:  Body function and structure: SF-36 physical dimension (sum score)  Activity and participation: SF-36 mental dimension (sum score)  Contextual factors: BSHS-B subscale affect & relations (sum score)  Both questionnaires (BSHSB & SF-36) were applied before and after rehabilitation and at 3- and 12-months follow-up. SF-36 dimensions were chosen as main variables because improving quality of life is an important aim of rehabilitation. Both dimensions correlate highly with physical and mental impairment and its improvement. The BSHS-B and its subscale “affect and relations” were chosen to evaluate changes in relationships due to burn injury. The BSHS-B is a burn specific instrument, which measures the squeals of a burn injury.  Further variables for pre-post and follow-up comparison are the physical outcome measures for strength and fitness (PWC, Grip strength (sum score), muscle strength (sum score)), scar assessment (Cutometer (R0), Vancouver Scar Scale (sum score)) and scare contracture and mobility (range of motion (single value of the most affected joint)). BSHS-B subscale simple abilities and hand function (sum scores), DASH (sum score) and LEFT (sum score) are also evaluated in pre-post and follow-up comparison regarding information about participation and activity. Variables regarding contextual and personal factors are the SCL-9 (screening instrument for psychopathologic symptoms, sum score), IES-R (screening instrument for symptoms of posttraumatic stress, sum score) and FsozU-14 (questionnaire to measure perceived social support, sum score).  Secondary outcome of the study refers to the comparison between the two centers (A and B) assuming non-inferiority. Outcome measures which are compared are the SF-36 physical dimension (sum score), the SF-36 mental dimension (sum score) and BSHS-B subscale “affect and relations”.  Third outcome of the study refers to the rehabilitation goals of the patients. Qualitative analysis of the goals is planned, as well as linkage to ICF categories. |
| Participant timeline  (page 8 of the manuscript:  Tab.3) | 13 | A total of 4 years is planned for realization of this study. Measurements are taken on admission, at the end of the rehabilitation program and on follow up after 3 and 12 months after discharge. Enrolment to the study ends after 3 years.  Tab. Time of assessments  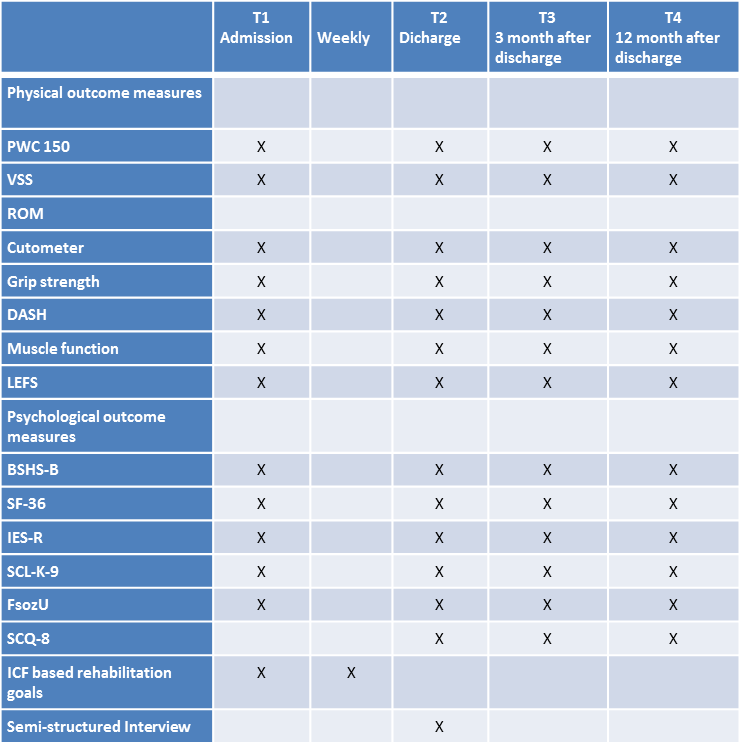 |
| Sample size  (page 9 of the manuscript:  Sample size) | 14 | A sample size of 162 patients is needed, N = 81 per center. Assuming a drop-out rate of 20% usually expected in a clinical trial, 92 patients per center would be needed.  We calculated the required case number for paired unilateral t-tests for the pre-post comparisons. Due to the small intervals between the measurements, a small to medium effect size of 0.25 with an α-error probability of 0.05 is expected. An expected correlation of the measured values of at least 0.60 requires a power of 0.80. The power analysis revealed a sample size of 162 patients. |
| Recruitment | 15 | The rehabilitation program was presented to referring clinics and at burn-specific congresses to administer patients. |
| **Methods: Assignment of interventions (for controlled trials)** | | |
| Allocation: |  |  |
| Sequence generation | 16a | Not applicable. No randomization of the intervention is planned. |
| Allocation conceal-ment mechanism | 16b | Not applicable. No randomization of the intervention is planned. |
| Implementation | 16c | Not applicable. No randomization of the intervention is planned. |
| Blinding (masking) | 17a | Not applicable. No randomization of the intervention is planned. |
|  | 17b | Not applicable. No randomization of the intervention is planned. |
| **Methods: Data collection, management, and analysis** | | |
| Data collection methods  (page 6-8 of the manuscript: Outcome and page 10: statistical methods) | 18a | Data will be collected on prepared case report form (CRF) and by questionnaires and will be entered contemporary into an Access database. The trial coordinator checks regularly on the database to promote data quality (plausibility – range of values; number of missing values, duplicates etc.).  Training for all raters with the cutometer was performed. A protocol for re-test acuity of the measure points was established and tested (Klimitz et al., in prep.). Furthermore, conduction of the semi-structured interview was trained.  **Description of study instruments**  Physical working capacity test 150 (PWC 150):  The participant is sitting on a mechanically braked bicycle ergometer (Ergofit Cardio-Line 3000). Heart rate is measured with a heart rate monitor with chest strap (Sigma Modell Classic, Sigma-Elektro, Neustadt/W, Germany). The participant is instructed to reach a pedal rotation rate of 70 rounds per minute (rpm). The test begins at 25 watts (W), workload is increased by 25W every 2min. When the individual target heart rate (150-age) is exceeded for more than 30s, the final workload stage will be recorded. A 5min recovery period with a workload of 25 W follows, heart-rate recovery is recorded (Noonann 2000). The PWC is a commonly used instrument for measuring physical fitness. Borg & Dahlström (1962) are reporting good reliability (intra test correlation r=0.97, re-test correlation after 8 month r=0.75) and validity.  PWC will not be performed if any contraindications are detected in the medical examination before the test. Test will be stopped if adverse symptoms (eg. dizziness, high pulse) are occurring. PWC index is calculated and a pre-post and pre-follow up comparison is made by t-tests (or if necessary by non-parametrical equivalence).  Muscle function test by Jamar digital hand dynamometer (Sammons Preston Inc., Bolinbrook, IL, USA):  Participants will be requested to press the dynamometer with maximum force. Three trials with each hand will be carried out. (Gittings 2016). Hamilton, McDonald and Chenier (1992) are reporting good reliability (r=0.82) and good validity.  This test will not be performed if any general contraindications are detected in the medical examination before the test. For each measurement – fist closure, finger tips grip and clamping grip – three measures are taken; averages are built and pre-post and pre-follow-up comparisons are analyzed be t-test (or non-parametrical equivalent).  Muscle function test by Janda (1959):  Major muscle function of the upper and lower extremities are tested according to Janda (1959) by a skilled physiotherapist, rating the muscle functioning from 0=”no contraction” to 5=”normal functioning”. A good reliability for manual muscle testing with correlation coefficients between 0.57 and 1.00 and good validity is reported (Cuthbert & Goodheart 2007). This test will not be performed if any general contraindications are detected in the medical examination before the test. Sum score will be built over all values and descriptive statistic for each time point will be performed.  Range of motion (ROM) of large joints:  A goniometer is used to measure the angle at the joints. Rooijen et al. 2016 are reporting an intra-class correlation coefficient of 0.85, a moderate up to perfect intra-rater reliability (0.68-0.98) and a good validity (r=0.82). This test will not be performed if any general contraindications are detected in the medical examination before the test. Values of the most affected joint will be chosen; difference values pre-post and pre-follow-ups will be built, z-transformed and then aggregated over patients. Descriptive statistic will be performed.  Vancouver scar scale:  The Vancouver scar scale (VSS) assesses four parameters of scar, including vascularization, pigmentation, pliability and height/thickness giving a range of 0–14 in the total score. The appearance of the burn scar will be evaluated by a physician (Baryza 1995). Internal consistency (0.71) and interrater reliability is acceptable (r=0.78) and convergent validity is good (Truong et al. 2007). Total scores will be built and pre-post respectively pre-follow-up comparison will be analyzed by t-test (or non-parametric equivalent).  Cutometer® MPA 580 (Courage + Khazaka):  The Cutometer® MPA 580 is a widely used device to measure the firmness and elasticity of skin. A negative pressure is created by vacuum and the skin is drawn into the aperture of the probe (6mm) and released after a defined time. Inside the probe, height of skin that is drawn up is determined by a non-contact optical measuring system. The resistance of the skin to the negative pressure (firmness) and its ability to return into its original position (elasticity) are displayed as curves (penetration depth in mm/time) and parameters are calculated (R0 and R2) (Nedelec 2014). Reliability is acceptable (>0.89), validity (correlation with VSS) is significant. No side effects of the cutometer were reported, it is widely used for scar assessment and cosmetic purposes. Measures for our study will all be performed by a physician and only applied on healed skin. For R0 pre-post respectively pre-follow-up comparison will be analyzed by t-test (or non-parametric equivalent).  The Disabilities of the Arm, Shoulder and Hand (DASH; German version, German et al. 2003):  The DASH is a 30-item self-report outcome measurement for physical function and symptoms in patients with one or multiple musculoskeletal disorders of the upper limb. In addition to the Disability/Symptom module (30 items, scored 1-5) it contains two optional modules: sports, performing Arts and work (each scale consists of four items, scored 1-5). Each score is transformed into a 0-100 scale. Higher scores indicate greater disability. The DASH is widely used and has demonstrated its clinical value for several different medical conditions of the upper limb. Test-retest reliability was r=0.90, internal consistency α=0.96 and strong correlations between DASH and other relevant well validated instruments were found. Total scores will be built and pre-post respectively pre-follow-up comparison will be analyzed by t-test (or non-parametric equivalent).  Lower Extremity Functional Scale questionnaire for Patients with foot and / or leg injuries (LEFS):  This questionnaire is a 20-item condition-specific functional status measure applicable to a wide spectrum of patients with lower extremity conditions of musculoskeletal origin. Each LEFS item is scored on a Likert scale from 0 to 4 with higher scores representing higher functional levels, the maximum score being 80 (Binkley 1999). Reliability (ICC 0.98) and internal consistency (CA 0.95) are good. Validity for the German version proved also to be good with correlation coefficients between r=0.52-0.919 (Naal et al. 2014). Total scores will be built and pre-post respectively pre-follow-up comparison will be analyzed by t-test (or non-parametric equivalent).  Burn-specific health scale brief questionnaire (BSHS-B; German Version, Stolle 2018):  The BSHS-B measures the most important domains of a patient’s quality of life which are affected after burn injury. It consists of 40 items divided into 9 different subscales: Affect, interpersonal relationships, sexuality, simple abilities, hand function, work, heat sensitivity, treatment regimens, and body image. Each item asks about the extent of a specific limitation and can be answered on a 5-point Likert- Scale that ranges from 0 = “extremely limited” to 5 = “not limited at all.” The higher the score, the lower the perceived impairment. The German version showed good psychometric properties. Cronbach’s alphas ranged from α=0.80-0.92, test-retest reliability ranged from r=0.72-0.97. Validity was proven with correlations of the subscales with relevant well-validated instruments to be good with correlation coefficients between r=0.30 - -0.77). Total scores and scale scores will be built and pre-post respectively pre-follow-up comparison will be analyzed by t-test (or non-parametric equivalent).  Short Form Health Survey (SF-36, German Version):  The SF-36 questionnaire is one of the most used assessments to measure health-related quality of life in adults. The questionnaire includes eight scales, four can be summarized into the General Mental Health scale, while the remaining four domains form the General Physical Health scale. All subdomains are assessed using a five-point Likert-Scale (except Physical Functioning which uses a three-point scale). Most subscales of the SF-36 are showing good internal consistency with α>0.70; only the scales General Health Perception and Social Role Functioning are lower (α=0.57 and α=0.69). Correlation coefficients for test-retest measures are lying between r=0.67 – 0.85. Correlation coefficients with relevant well-validated other scales are between r=0.44-0.75. Total scores and scale scores will be built and pre-post respectively pre-follow-up comparison will be analyzed by t-test (or non-parametric equivalent).  Symptom-Checklist-9 (SCL-9-K, Sereda 2016) :  The original Symptom Checklist-90-Revised (SCL-90-R) is a widely used questionnaire developed to determine a number of psychological symptoms. Its shortened version showed equally high internal consistencies as suitable screening instruments to assess a wide range of psychopathologic symptom. The questionnaire contains one-dimensional items which are scored on a 4-point Likert scale ranging from 1 (not at all) to 4 (extremely). Internal consistency is good (α=0.89). Correlation between long (SCL-90) and short version (SCL-9-K) is high (r=0.91). Total score will be built and pre-post respectively pre-follow-up comparison will be analyzed by t-test (or non-parametric equivalent).  Impact of Event Scale-Revised (IES-R, Horowitz 1979):  The IES-R is a 22-item, self-report questionnaire that assesses posttraumatic stress symptoms in relation to a specific event. It is one of the most commonly used metrics of posttraumatic stress disorder symptomatology. Cronbach’s α shows good internal consistency (α=0.89), correlation coefficient between the short and the long form is high (r=0.91). Total score will be built and pre-post respectively pre-follow-up comparison will be analyzed by t-test (or non-parametric equivalent).  Recording perceived social support (F-SozU-14, Fydrich 2009):  The perceived social support will be measured with the short version of the Freiburg Social Support Questionnaire (F-SozU-14), a 14-item questionnaire comprising emotional and practical support as well as social integration and social burdens. Cronbach’s α shows good internal consistency (α=0.91) and test-retest reliability (r=0.96). Total score will be built and pre-post respectively pre-follow-up comparison will be analyzed by t-test (or non-parametric equivalent).  Patient Satisfaction Questionnaire (CSQ-8, Schmidt 1989):  At discharge and after 3 and 12 months, patients were asked to complete the German version of the Client Satisfaction Questionnaire-8 (CSQ-8). The CSQ-8 is a unidimensional, 8-item, questionnaire assessing patient satisfaction with the rehabilitation. Cronbach’s α shows good internal consistency (α=0.87-0.93). Correlation coefficients with other relevant well validated scales are showing good validity (r=0.60-0.80). Total score will be built and pre-post respectively pre-follow-up comparison will be analyzed by t-test (or non-parametric equivalent).  Rehabilitation goals:  Participants are questioned at the beginning of the rehabilitation about their primary goals they want to reach during the course of rehabilitation. Once a week they as well as the therapists were asked to fill out a questionnaire about their satisfaction with goal achievement. Further they could express if their goals for the following week have changed. These formulated goals will be linked to the ICF categories. Qualitative analysis will be performed.  Semi-structured feedback interview  At the end of the rehabilitation program a semi-structured feedback interview will be held in which the patients are asked to describe their experiences with the rehabilitation program by a trained psychologist who was not involved in the rehabilitation process. Qualitative analysis will be performed. |
| (page 8 of the manuscript: Outcome Tbl 3) | 18b | Participants retention:  Assessments are performed pre and post inpatient rehabilitation. They are associated with the normal clinical assessment routine before and after rehabilitation treatment. Therefore, we expect a low dropout rate.  At discharge and the first follow-up participants are informed about the next appointment. In addition, patients are getting an invitation by mail 3 Weeks bevor the appointment.  Outcome data for participants who discontinue:  Outcome data of patients participating only in pre-post assessments (T1/ T2) will be fully part of analysis, except participants retract their consent for usage of the data. If participants decline study participating after the first assessment (T1) data are not used, besides of demographic Data (sex, age) and total of affected body surface, in order to check for a systematic bias. |
| Data management  (not mentioned in the manuscript) | 19 | Data entry and quality:  For data entry a digital form was created with Access. For each variable, input characteristics are defined such as string or numeric, length of input and decimal or not, in order to minimize failures during data entry. On a regularly basis data will be checked with SPSS about double entries, displaying maximum and minimum values and frequency of missing values. Date entry will be performed contemporary by a research assistant therefore errors during completing of the CRF will be noticed soon. In case of an error the research assistant will confer with the clinician who filled in the form (4-eye-principle).  Data security:  CRF’s, questionnaires and the data base contain a numeric code instead of patient names. CRF’s and questionnaires are kept in a locked filing cabinet to which only the project leader, study coordinator and the research assistant have access. The access database is kept on a laptop without internet access, which is also kept in a locked cabinet, only staff of the project have access.  A coding list is kept for assignment at follow-up, only project manager, study coordinator and research assistant have access. The coding list is kept apart from CRF’s, questionnaires and the data base.  All information can also be found in the study protocol. |
| Statistical methods  (page 10 of the manuscript: Statistical methods) | 20a | For statistical analysis data will be checked for normal distribution. Data are expressed by means with standard deviations and medians with interquartile range as appropriate. Categorical variables will be expressed as percentages.  Pre-post comparison (T1 – T2) of the outcome measures will be tested by paired one-sided t-tests. Longitudinal treatment effects will be tested by paired one-sided t-tests between T1 and T3 respectively T4.  The non-interiority between the two centers will be proved with a power of 0.80 if differences are smaller than 0.4 standard deviations. Since randomized assignment to the centers is not possible, a potential selection bias between the samples is possible. In that case samples will be matched by age, gender, VKOF, ABSI and inhalation trauma along the procedure of propensity score matching. The statistical evaluation will be carried out in cooperation with the statistician who participated in the design. |
| (page 10 of the manuscript: Statistical methods) | 20b | Descriptive analysis of the patients’ rehabilitation goals and their linkage to the ICF categories will be made. Patient’s and therapist’s assessments of goal achievement will be compared.  Results of the CSQ-8 will be expressed by means and standard deviation.  The results of the semi-structured interviews will be evaluated qualitatively. |
| (page 10 of the manuscript: Statistical methods) | 20c | Only datasets with a minimum of an assessment bevor and after the rehabilitation will be part of the analysis. Cases of treatment non-adherence (e.g. concomitant care, to skip training sessions) will be kept for analysis.  A drop-out rate of 20% usually expected in a clinical trial is calculated in the sample size. But we expect a lower drop-out rate of 10%, as patients are usually closely connected to the burn centers. Drop-out patients as well as non-participants will be assessed with age, sex, TBSA and checked for systematic differences with the study population.  In questionnaires, we will allow a maximum of 10 % missing data, which will be replaced by imputation, but only if 50% missing data are not exceeded per scale. No missing data are expected in the CRFs, as the completeness is checked immediately after data entry. |
| **Methods: Monitoring** | | |
| Data monitoring  (not mentioned in the manuscript) | 21a | The data monitoring committee (DMC) is composed of members of the participating institutions, members of the sponsor and independent external experts. The DMS meets annually to review the progress and compliance with the timeframe. Interim results will be presented at the annual meeting. A written report is submitted to the sponsor every year.  Beside of control of the time frame the sponsor is not involved in data collection or monitoring. |
| (page 9 of the manuscript: interim analysis and stopping rules) | 21b | Termination of the study is planned, if severe side effects of the treatment occur, responsibility for this decision rests with the senior and junior leaders of the study (sponsor is to be informed). For single patient termination of the rehabilitation treatment because of severe side effects and therefore termination of its participation in the study will be decided by the rehabilitation team.  Further, termination of the study is planned, if it’s foreseeable that the planned sample size will not be reached during the enrolment. Sponsor and senior leader are responsible for this decision.  Interim analysis of the data will be performed annually and reported to the data monitoring committee, the sponsor and the members of the research group. |
| Harms  (page 5 of the manuscript: Interventions | 22 | Only standard treatment for inpatient rehabilitation is planned, no new interventions will be applied. Solicited and spontaneously reported adverse events or unintended effects of the standard treatment will be recorded during the rehabilitation in the clinical files of the patients. Treatment will be changed, and study participation ends. These cases will also be documented in the CRF’s of the patients and reported to the sponsor, the study team and the data monitoring team. A report of adverse events will be also part of publication.  At the follow-up assessments no treatments will be applied. Should there be any adverse effects due to the assessment for the study, patient will be administered immediately to the doctor of duty. |
| Auditing  (not mentioned in the manuscript) | 23 | No external auditing of the trial is planned.  CRF’s will be proofed regularly for completeness and plausibility during data entry. |
| Ethics and dissemination | | |
| Research ethics approval  (page 12 of the manuscript: Ethical approval) | 24 | The study is approved by the federal ethics committee of Rhineland-Palatinate. Participants must give written consent prior of inclusion to the study. |
| Protocol amendments  (not mentioned in the manuscript) | 25 | In case of trial modification this will be reported to the ethics committee, from which approval is obtained formally. Further, the clinical trial register will be informed about changes, as well as the sponsor and the investigation team. |
| Consent or assent  (page 2 of the manuscript: Tab 2 (..written consent..)) | 26a | Potential participants of the study will be given detailed information about the trial in written form as well as orally by one of the research members. If they are willing to participate, informed consent will be obtained by the senior or junior study leader. The consent will be signed by the participant and by the study leader. The participant will receive a copy. The original will be archived by the senior study leader. No under aged persons or not competent persons will be included in the trial. |
|  | 26b | Data collected will not be used in ancillary studies or promoted to another party. |
| Confidentiality  (not mentioned in the manuscript) | 27 | Information for the study will be collected on CRF’s and questionnaires which contain a numeric code. Due to assignment of the data of the different assessment points during the study a code list will be maintained. This list will be kept strictly separate to the CRF’s and the database in a locked cabinet, only the project leader, study coordinator and the research assistant have access.  Informed consents of the participants will be stored apart from other study documents in a locked file cabinet at each study center, only the project leader of the center will have access.  The anonymized database will be kept on a laptop without internet access, which is also kept in a locked cabinet, only staff of the project have access. For joint analysis data of center B must be transferred to the coordinating center. The anonymized data set will be transferred by encrypted file on a stick (protected by password) by registered mail.  After finishing the enrolment and study assessment study data will be stored 10 years (along data privacy act of Germany), after that period the files will be irretrievably destroyed. |
| Declaration of interests  (page 12 of the manuscript: Competing interests) | 28 | There are no financial or competing interests of the investigators for the study. |
| Access to data  (not mentioned in the manuscript) | 29 | The investigators involved have access to the anonymized dataset. The sponsor will be allowed to view the anonymized dataset and the protocol of the analysis. The data will be deleted after a retention period of 10 years. |
| Ancillary and post-trial care  (not mentioned in the manuscript) | 30 | Participant insurance has been set up with 500.000€ per participant and 5.000.000€ for the total study. |
| Dissemination policy  (not mentioned in the manuscript) | 31a | The trial results will be published in corresponding journals and conferences to healthcare professionals, the public. The sponsor will receive a final report. |
|  | 31b | At publication of the study only authors are named, which will have substantial contributions to the study design, patients’ enrolment and assessment, data management and analysis.  No use of professional writers is planned. |
|  | 31c | The full study protocol, the CRF’s and the statistical code can be requested by interested fellow researchers from the senior study leader. No public access to the participant-level dataset is planned. |
| Appendices |  |  |
| Informed consent materials | 32 | Information about the study  Consent form |
| Biological specimens | 33 | Not applicable. There is no collection of biological specimens. |
